# Cognitive decline and brainstem hypometabolism in long COVID: A case series

**DOI:** 10.1002/brb3.2513

**Published:** 2022-03-15

**Authors:** Jacques Hugon, Mathieu Queneau, Marta Sanchez Ortiz, Eva Flore Msika, Karim Farid, Claire Paquet

**Affiliations:** ^1^ Center of Cognitive Neurology University of Paris Lariboisière FW Hospital, APHP, Inserm 1184 Paris France; ^2^ Memory Clinic Paris France; ^3^ Department of Molecular Imaging North Cardiologic Center Saint Denis France; ^4^ Department of Molecular Medicine CHU Fort de France Martinique France

**Keywords:** brainstem lesions, cerebral FDG PET, cognitive deficits, long COVID

## Abstract

**Objective:**

To assess FDG cerebral PET in patients suffering from cognitive impairment linked to Long COVID. The COVID pandemic has affected dozens of millions of people around the world and has resulted in the deaths of more than 3 million people. Following the acute forms, it has been reported sometimes long forms of COVID, with involvements of several organs including the brain. Neurological complications can include cognitive disturbances (brain fog) that are very common and can seriously disturb the life of patients.

**Methods:**

Fluorodeoxyglucose PETs were performed in 3 patients with cognitive decline following COVID infection.

**Results:**

We report here 3 cases of brain fog with major hypometabolic areas of the pons revealed by the cerebral FDG PET.

**Conclusion:**

The dysfunction of the locus coeruleus in these patients could partly explain the cognitive disorders observed. Further studies involving larger cohorts of patients suffering from cognitive dysfunction will be needed to determine if the brainstem is frequently affected in these patients.

## Graphical abstract

Cognitive deficits linked to long COVID can be associated with brainstem hypometabolic areas on the FDG PET scan.

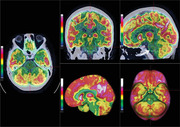

## 1 INTRODUCTION

The COVID pandemic that began in December 2019 in China in Wuhan has affected dozens of millions of people around the world and has resulted in the deaths of several million people (Dong et al., 2020). The clinical complications observed during SARS‐COV‐2 infections are diverse and can involve the lungs, heart, kidneys, muscles, nervous system, and can lead to long‐term fatigue (Nalbandian et al., 2021). The neurological complications observed after severe forms of infections can include strokes, confusions, and damage to the cortico‐spinal tract (Helms et al., 2020; Nalbandian et al., 2021). A few cases of Guillain Barré syndrome have been reported as well as frequent cases of anosmia and ageusia (Ellul et al., 2020).

In a recent study including 236,379 patients, neurological and psychiatric complications were evaluated at 33.62% after 6 months of patient follow‐up and included strokes, cerebral hemorrhages, Parkinsonism syndromes, dementias, anxiety, and depressive syndromes (Taquet et al., 2021). Neurological complications were more frequent in patients admitted to ICU than in non‐admitted patients. Another report of 100 patients with COVID infection who were not hospitalized found long‐term neurological complications (Graham et al., 2021). The mean duration of follow‐up was 4.72 months. The mean age was 43.2 years old and 70% were female. The most common neurological complication was cognitive complaints (brain fog) (81%) followed by headache (68%), paresthesia, (60%), anosmia, and ageusia. Few studies have analyzed long‐term cognitive complications in patients with mild‐to‐moderate forms of COVID infection. We report here the study of three patients with cognitive complaints after COVID infection; two being not admitted for their COVID infection. The most striking result is the presence of hypometabolic zones on cerebral FDG PET, isolated or not, at the level of the brainstem and in particular at the level of the pons.

## 2 PATIENTS AND METHODS

We included the cases of three long COVID patients with cognitive complaints who were explored at the Center of Cognitive Neurology at Lariboisière Hospital or at the Memory Clinic 75008 in Paris from February to May 2021.

### 2.1 PET data processing

PET data were acquired using either of three PET/CT scanners (Discovery MI4, IQ5 and IQ4, GE Healthcare, Milwaukee, USA). PET data acquisition followed a standard protocol, which consisted in 20 min of resting with sensory deprivation (silent and dark room, eye mask), 18FDG injection (2 MBq/kg), 30 min of additional resting, PET data acquisition (15 min), and CT for attenuation correction. Images were reconstructed using standard procedures (iterative reconstruction algorithm, attenuation correction using CT‐based attenuation map).

### 2.2 18FDG‐PET analysis

**FIGURE 1:**
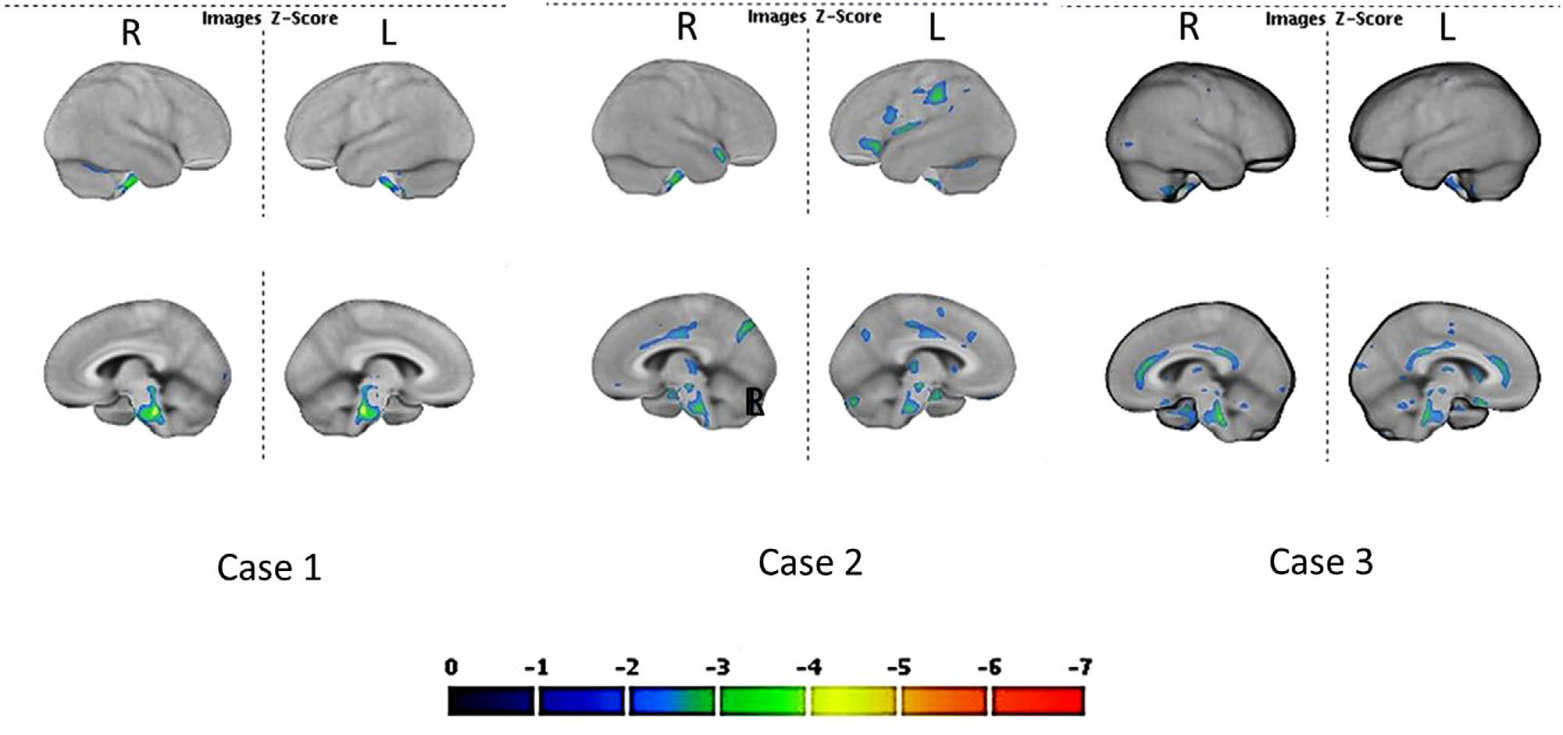
The results of the FDG PET imaging in the three patients. In patient 1, only the pons was mainly hypometabolic. In patient 2, in addition to the pons, the precuneus and the left parietal areas were hypometabolic. In patient 3, the pons and the cingulate cortex were found to be hypometabolic

**FIGURE 2:**
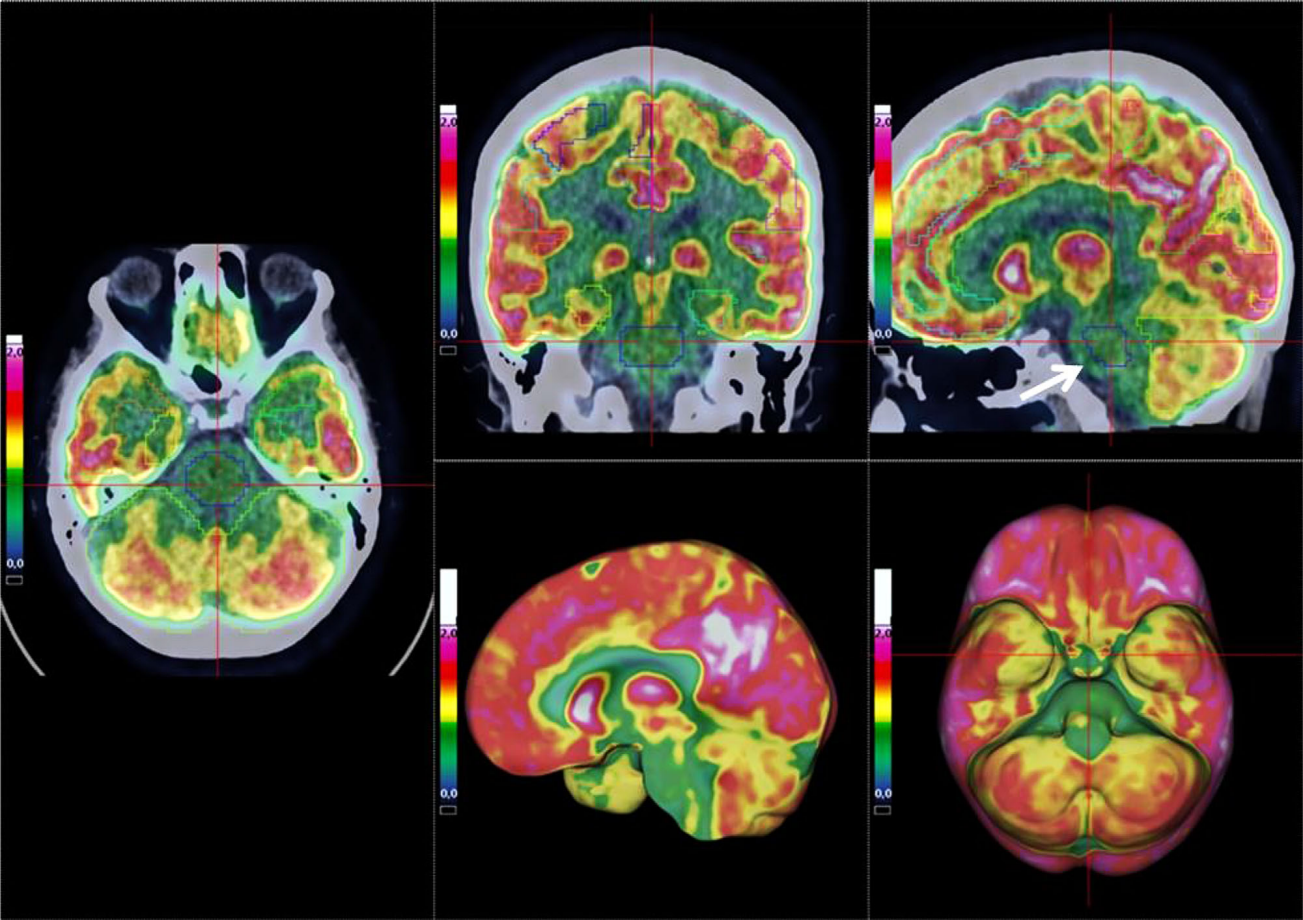
Case 1: These figures show the hypometabolic region of the brainstem (arrow)

A quantitative ROI‐based analysis using CortexID suite (GE Healthcare), a fully automated spatial normalization applied to brain PET data, was performed. A set of 26 VOIs of particular interest in neurodegenerative conditions and based on functional anatomy generate individual maps of cortical hypometabolism, according to the ICBM152 Atlas. Results were automatically compared with those of a set of 294 healthy controls matched for age and sex. The results are expressed as Z‐scores, i.e., the difference divided by the standard deviation. Hypometabolic regions with more than two standard deviations are shown in Figures 1 and 2.

### 2.3 Case 1

A 65‐year‐old woman was hospitalized in ICU in March 2020 with COVID infection and acute respiratory failure. She was put on artificial ventilation for several days. She had type 2 diabetes, hypertension, hypercholesterolemia, and chronic respiratory failure. PCR was positive. She suffered from myocarditis during her chronic hospitalization and hallucinations after her discharge. A few weeks later, she complained of memory problems, poor concentration, and instability when standing. Neurologic examination only shows extrapyramidal hypertonia of the right upper limb. The brain MRI was normal. MMSE was 29/30. The complete neuropsychological examination shows mainly executive and memory impairments.

### 2.4 Case 2

A 70‐year‐old hypertensive man was diagnosed with COVID infection in April 2020 describing fever, dyspnea on exercise, and sweating that went away within 2 weeks. The patient was not hospitalized. PCR was positive. In June 2020, the patient complained of progressive cognitive disorders. Neurological exam and brain MRI were normal in November 2020. MMSE was 21/30. The complete neuropsychological assessment showed memory disorders, executive, and language deficits.

### 2.5 Case 3

A 42‐year‐old woman had a COVID infection in March 2020 with fever, myalgia, dyspnea, and anosmia. She was not hospitalized and recovered within 3 weeks. She had no pathological history. COVID PCR was negative, but COVID serology was positive when she was examined in July 2020 for cognitive complaint. Neurological exam and brain MRI were normal. MMSE was 25/30. The complete neuropsychological evaluation revealed working memory deficit and abnormal executive functions.

All patients were proposed a long‐term cognitive rehabilitation.

## 3 RESULTS

The results of the FDG PETs are shown in Figure 1. In Case 1, hypometabolic regions were mainly localized in the pons. In Case 2, mild hypometabolic regions were depicted in the left parietal and precuneus areas, but the pons was markedly hypometabolic, as well as the medial temporal lobe. In Case 3, the pons was clearly hypometabolic, whereas anterior and posterior cingulate cortex were less affected, the medial part of the orbitofrontal cortex seems also impaired and also the medial temporal lobe. In these three long COVID patients with cognitive deficits, the main involved regions included the pons especially in Case 1 (Figure 2), whereas other regions were mildly involved on FDG PET. It is important to notice that patients 2 and 3 were not hospitalized, meaning that the severity of hypoxia was less pronounced than in Case 1.

## 4 DISCUSSION

The main interesting finding of these cases is that long COVID marked by cognitive complaints and deficits can be associated at least with hypometabolic regions of the brainstem and more especially of the pons. In Cases 2 and 3, other hypometabolic regions detected with FDG PET scans could have also contributed to the clinical symptoms. Two of the patients had mild forms of COVID infection and were not hospitalized, suggesting that long‐term brainstem lesions may be direct or indirect consequences of SARS‐COV‐2 brain invasion in mildly affected individuals.

The first question that can be addressed is whether the brainstem is affected in COVID cases. A recent neuropathological report has included 43 patients who died in hospitals, nursing homes, or at home (Matschke et al., 2020). The results of this histological study revealed that activated microglia and cytotoxic T lymphocytes were more detected in the brainstem and cerebellum, suggesting that inflammatory processes could occur more severely in these regions. No direct evidence of SARS‐Cov‐2‐induced brain lesions were noted. Brainstem inflammatory lesions were the most common results of this neuropathological data, and this finding could partly explain the hypometabolic areas observed with FDG PET in the reported cases.

A recent study has displayed two cases of hospitalized patients with COVID infection and suffering from neurological complications. FDG PET results revealed hypometabolisms of the olfactory/rectus gyrus accompanied by diffuse hypometabolisms of the amygdala, the hippocampus, the cingulate cortex, the thalamus, the cerebellum, and the pons (Guedj et al., 2021). The authors suggest the hypothesis of a SARS‐Cov‐2 neurotropism via the olfactory bulb spreading to other brain regions including the brainstem. The involvement of the brainstem has also been recently described in adults and children in other reports (Guedj et al., 2021; Manca et al., 2021; Morand et al., 2021). Guedj et al. suggested a possible role of angiotensin converting enzyme (ACE) receptors as an olfactory gateway for this neurotropism.

In addition, we have recently shown that, in long COVID patients, cognitive impairment in hospitalized and non‐hospitalized individuals could be associated with hypometabolic areas of the cingulate cortex without affecting the brainstem (Hugon et al., 2021).

The second question that could be addressed is how the brain stem involvement could explain cognitive impairment in long COVID patients? One of the important nucleuses located in the brainstem/pons is the locus coeruleus. It has long been recognized that the locus coeruleus could play a clear role in the process of cognition (Berridge & Waterhouse, 2003). Neurons of the locus coeruleus are the major source of noradrenaline in the brain, and noradrenaline is a neuromodulatory neurotransmitter playing a key role in the control of forebrain activities (Sara, 2009). The locus coeruleus nucleus has many projections including to the prefrontal cortex and the hippocampus involved in the modulation of cognition. In addition to its action on sensory processing and walking behavior, noradrenaline contribute to various aspects of cognition including attention, behavioral flexibility working memory, and long‐term memory (Borodovitsyna et al., 2017). The loss of noradrenaline could also be implicated in cognitive diseases like Alzheimer's disease (Holland et al., 2021).

In conclusion, these findings could suggest that cognitive deficits observed in these COVID patients could partly originate from the dysfunction of the locus coeruleus. Our results also emphasize the need to explore COVID patients complaining of cognitive deficits with cerebral FDG PET even in non‐hospitalized subjects affected by mild Sars‐Cov‐2 infections.

## FUNDING

None.

### PEER REVIEW

The peer review history for this article is available at https://publons.com/publon/10.1002/brb3.2513

## Data Availability

The data that support the findings of this study are available on request from the corresponding author.
